# PP76 Clinical Guidelines For Anti-Neutrophil Cytoplasmic Antibodies-Associated Vasculitis: Incorporating Rituximab To Enhance Equity In Access And Treatment In Brazil

**DOI:** 10.1017/S0266462325102948

**Published:** 2025-12-29

**Authors:** Aline Rocha, Nitza Ferreira Muniz, Ana Carolina Pereira Nunes Pinto, Álvaro Atallah

## Abstract

**Introduction:**

Anti-neutrophil cytoplasmic antibodies (ANCA)-associated vasculitis is a rare disease with high morbidity and mortality rates, posing challenges for incorporating treatments due to limited high-quality evidence. Brazil recently developed a Clinical Protocol and Therapeutic Guideline (PCDT) after incorporating rituximab into the public healthcare system. This study explored the development of a Technical Scientific Report (TSR) addressing rituximab’s effectiveness and costs as well as stakeholder participation in shaping equitable, evidence-based recommendations.

**Methods:**

A TSR was initially developed that included an evaluation of evidence, an economic model, and a budget impact analysis. Cyclophosphamide, which is already available in the public health system, was used as the comparator. Based on this analysis, the National Committee for Health Technology Incorporation (CONITEC) issued a preliminary recommendation against incorporating rituximab for ANCA-associated vasculitis. This TSR was submitted for public consultation (6 to 25 April 2023), during which community contributions were received. All submissions were thematically analyzed, and relevant contributions were re-evaluated. The updated findings were then presented to CONITEC for a final decision, incorporating new perspectives and refined results.

**Results:**

During the public consultation, 169 contributions were received: 56 from patients and caregivers unanimously supporting rituximab’s incorporation and 113 technical submissions highlighting cyclophosphamide’s toxicity and lack of response in 25 percent of patients, and rituximab’s economic benefits. Revised economic analyses found a reduced incremental cost-effectiveness ratio when comparing rituximab with intravenous cyclophosphamide and clarified the five-year budget impact. These updates, along with strong patient participation, influenced the reversal of CONITEC’s initial recommendation, leading to rituximab’s approval in June 2023 and the development of the PCDT.

**Conclusions:**

The incorporation of rituximab into Brazil’s public health system and the creation of the PCDT for ANCA-associated vasculitis represents a significant advancement in treating rare diseases and may help promote remission, reduce relapses, and improve patient quality of life. By integrating patient perspectives and evidence-based practices, the PCDT strengthens equity and access, fostering comprehensive care for rare diseases in Brazil.

